# An Analysis of Enzyme Kinetics Data for Mitochondrial DNA Strand Termination by Nucleoside Reverse Transcription Inhibitors

**DOI:** 10.1371/journal.pcbi.1000261

**Published:** 2009-01-09

**Authors:** Katherine V. Wendelsdorf, Zhuo Song, Yang Cao, David C. Samuels

**Affiliations:** 1 Virginia Bioinformatics Institute, Virginia Polytechnic Institute and State University, Virginia, United States of America; 2 Department of Computer Science, Virginia Polytechnic Institute and State University, Virginia, United States of America; 3 The Center for Human Genetics Research, Department of Molecular Physiology and Biophysics, Vanderbilt University Medical Center, Nashville, Tennessee, United States of America; Lilly Singapore Centre for Drug Discovery, Singapore

## Abstract

Nucleoside analogs used in antiretroviral treatment have been associated with mitochondrial toxicity. The polymerase-γ hypothesis states that this toxicity stems from the analogs' inhibition of the mitochondrial DNA polymerase (polymerase-γ) leading to mitochondrial DNA (mtDNA) depletion. We have constructed a computational model of the interaction of polymerase-γ with activated nucleoside and nucleotide analog drugs, based on experimentally measured reaction rates and base excision rates, together with the mtDNA genome size, the human mtDNA sequence, and mitochondrial dNTP concentrations. The model predicts an approximately 1000-fold difference in the activated drug concentration required for a 50% probability of mtDNA strand termination between the activated di-deoxy analogs d4T, ddC, and ddI (activated to ddA) and the activated forms of the analogs 3TC, TDF, AZT, FTC, and ABC. These predictions are supported by experimental and clinical data showing significantly greater mtDNA depletion in cell culture and patient samples caused by the di-deoxy analog drugs. For zidovudine (AZT) we calculated a very low mtDNA replication termination probability, in contrast to its reported mitochondrial toxicity *in vitro* and clinically. Therefore AZT mitochondrial toxicity is likely due to a mechanism that does not involve strand termination of mtDNA replication.

## Author Summary

While HIV/AIDS therapy is very successful at controlling HIV infection, the therapy must continue for the remainder of the patient's life. Approximately one-fourth of these patients suffer from serious drug toxicity problems. It is generally believed that the toxicity of these drugs is caused by damage to mitochondria, the “power plants” of every cell. But we do not know exactly how this damage occurs. The most common explanation is that these drugs damage mitochondria in the same way that they control the virus, by interfering with DNA replication. We tested that idea by analyzing data for the interaction of several AIDS drugs with the mitochondrial DNA polymerase, the protein responsible for copying mitochondrial DNA. By using a detailed simulation of the mitochondrial DNA polymerase, we show that some of these drugs do interact well enough with the mitochondrial DNA polymerase to lead to toxic effects. However, many of these drugs, including the commonly used drug AZT, had very little toxic effect in this simulation although AZT often causes toxicity in patients. This indicates that the toxicity of AZT occurs through some other process and not through the direct interruption of mitochondrial DNA replication.

## Introduction

Current guidelines for highly active anti-retroviral treatment (HAART) regimens of HIV-positive patients recommend two drugs of the nucleoside reverse transcriptase inhibitor (NRTI) class (Table 1) [1]. This class currently consists of: stavudine (d4T), lamivudine (3TC), zidovudine (AZT), zalcitabine (ddC), didanosine (ddI), abacavir (ABC), emtricitabine (FTC) and tenofovir (TDF, a nucleotide analog). Though zalcitabine (ddC) at the time of this writing is still technically approved for treatment its distribution in the United States was discontinued by Roche in 2006. In their activated tri-phosphorylated forms, each NRTI acts as a nucleotide analog interacting with the HIV viral reverse transcriptase as an alternative substrate to the natural nucleotides [2],[3]. Each of these analogs lacks the 3′ OH group necessary for incorporation of the next nucleotide thereby terminating viral DNA strand elongation. Although NRTIs are effective drugs and have helped usher HIV into the category of a controllable chronic disease, they are also often toxic, inducing side effects such as lactic acidosis, neuropathy, nausea, lypodistrophy, and myopathy in patients. Intolerance of such side effects is a common reason for treatment discontinuation [4]. Any decrease in patient compliance to the treatment regimen is a serious concern that can lead to an increase in viral resistance and ultimately to treatment failure. The first step in ameliorating these side effects and preventing them in future antiviral treatments is to understand the mechanisms behind the mitochondrial toxicity of the NRTIs that are in use today. As we discuss below, many mechanisms of the mitochondrial toxicity have been proposed. In this paper we specifically consider the plausibility of the currently most widely accepted hypothesis for the toxicity mechanism for this class of drugs; interference of mitochondrial DNA replication by the activated drug.

**Table 1:** Nucleoside and nucleotide analogs used in this study.

Drug	Abbreviation	Natural Nucleoside	Comment
Abacavir	ABC	dG	The activated form is CBV-TP.
Acyclovir	ACV	dG	Used in treatment against Herpes viruses including HSV1 and 2, chickenpox and herpes zoster.
Didanosine	ddI	dA	Must be aminated to become ddA, which is activated form. The nonactivated form, however, has also been shown to incorporate into mtDNA.
Dideoxyadenosine	ddA	dA	The active form of ddI.
Emtricitabine	FTC(−)	dC	The unnatural enantiomer that is approved for treatment of HIV.
Emtricitabine	FTC(+)	dC	The natural enantiomer that is more toxic and not approved for treatment.
Lamivudine	3TC(+)	dC	This natural enantiomer of 3TC is not used in treatment, but is used in studies that look at effects of configuration on toxicity and efficacy.
Lamivudine	3TC(−)	dC	The unnatural enantiomer that is approved for treatment of HIV.
Stavudine	d4T	T	
Tenofovir	PMPA, TDF	dAMP	A nucleotide analog.
Zalcitabine	ddC	dC	Not currently recommended for clinical use.
Zidovudine	AZT	T	

### The Polymerase-γ Hypothesis

Polymerase-γ (pol–γ) is the only polymerase responsible for mitochondrial DNA replication. While pol-γ is not believed to directly regulate mtDNA levels, pathogenic mutations in the gene *POLG* do affect the stability of mtDNA and cause mtDNA depletion [5]. Polymorphisms found in the *POLG* gene in the human population may cause a natural variability in the activity of this complex enzyme and may conceivably play a role in patient variability in NRTI drug toxicities.

In a study conducted by Martin et al. [6] the approved NRTIs were shown to inhibit various host DNA polymerases. After the HIV Reverse Transcriptase, the highest affinity of the NRTIs was for polymerase-γ. This, along with the fact that many of the NRTI side-effects resemble symptoms of mitochondrial genetic disorders, implicated interaction with polymerase-γ and subsequent depletion of mtDNA as a potential cause of NRTI toxicity giving rise to the polymerase-γ hypothesis [7]. Indeed, experiments have demonstrated decreased mtDNA amounts in various tissue types of NRTI-treated HIV positive patients [8]–[11]. In addition, mtDNA depletion was observed in parallel with cell death, mitochondrial morphological changes, and increased lactate production in liver, heart, neuron, skeletal muscle, adipose, and blood cell cultures after incubation with different NRTIs [12]–[20]. The possible polymerase-γ dependent toxicity mechanisms that comprise the polymerase-γ hypothesis are (i) direct inhibition of polymerase-γ by NRTI-triphosphate without incorporation into the mtDNA, (ii) chain termination of mtDNA replication following incorporation of the NRTI triphosphate, and (iii) incorporation of the analog triphosphate into mtDNA without chain-termination allowing the NRTI to continue as a point mutation in mtDNA [21].

However, there also exists a substantial body of data that are not consistent with toxicity mechanisms resulting in depletion of mtDNA. Martin et al. [6] showed no association between inhibition of polymerase-γ by NRTIs and mtDNA depletion. Mitochondrial dysfunction has been observed *in vitro* in mouse muscle, white adipose, brain, liver, and heart tissue [22], hepatoma cell lines [20] as well as CD4 cells [19] after incubation with NRTIs although no significant decrease in mtDNA amount was observed. Particularly, incubation of liver and skeletal muscle cells with ddC, ddI, d4T, and AZT show a higher rate of lactate production in the presence of AZT, but the least amount of mtDNA depletion [15],[18]. In clinical settings mtDNA depletion has been seen in parallel with normal cytochrome c oxidase activity, a sign of correct mitochondrial function [23], and was not associated with lipoatrophy [24] (although that study measured mtDNA depletion in blood samples, not fat cells). Taken together, these findings indicate a weak relationship between mtDNA copy number and nucleoside analog toxicity. This warrants a deeper look at the data concerning the interaction of different NRTIs with polymerase-γ. To this end, we have simulated the DNA replication process of mitochondria. Using enzyme kinetics data gathered from Johnson et al. [25], Feng et al. [26], and Hanes et al. [27],[28] we have carried out a series of simulations of mtDNA replication in the presence of various nucleoside analogs that interact with polymerase-γ (Table 2). These simulations bridge the gap between the basic enzyme kinetics data and the probability of failure of the mtDNA replication process.

**Table 2:** Enzyme kinetics parameter values used for polymerase-γ interaction with each activated analog, in order of decreasing k_cat_/K_m_.

NRTI	K_m_ (µM)	k_cat_ (sec^−1^)	k_cat_/K_m_ (µM sec)^−1^	V_exo_ (sec^−1^)
ddC	0.041	0.660	16.1	0.00002
ddA	0.022	0.310	14.1	0.0005
d4T	0.045	0.24	5.33	0.0004
FTC(+)	0.79	0.84	1.1	0.0048
3TC(+)	1.5	0.35	0.23	0.02
Acyclovir	6	1.03	0.17	0.0021
ddI	6.3	0.15	0.024	0.0007
3TC(−)	9.2	0.125	0.01	0.015
TDF	40.3	0.21	0.005	0.0007
AZT_2001_	187	0.2	0.001	0.0004
ABC	13	0.0018	0.0014	0.0016
FTC(−)	62.9	0.0086	0.00014	0.0048
AZT_2007_	280	0.001	0.0000036	Not reported

Enzyme kinetics data taken from references [25]–[28].

## Methods

### The Drugs Included in this Study

Thirteen analogs were used in the simulations (Table 1). These included eight drugs of the NRTI class currently approved for human treatment- stavudine (d4T), lamivudine (3TC(−)), zidovudine (AZT), zalcitabine (ddC), didanosine (ddI) (whose active form is dideoxyadenosine (ddA) triphosphate), abacavir (ABC), emtricitabine (FTC(−)) and tenofovir (TDF) [1], and one anti-herpes drug, acyclovir (ACV). In addition we modeled the effects of the natural enantiomers of FTC(+) and 3TC(+) that have been used to explore a possible role of stereochemistry in the efficacy of strand termination [29], and ddI in its non-activated form. Since this study focuses on strand termination, we have not included FIAU, an anti-hepatitis B drug that tragically resulted in the deaths of five patients in phase 2 trials and whose toxicity is believed to be due to errors in mtDNA replication [30],[31], though not necessarily through strand termination [25],[31].

### Computational Model

**Figure 1:**
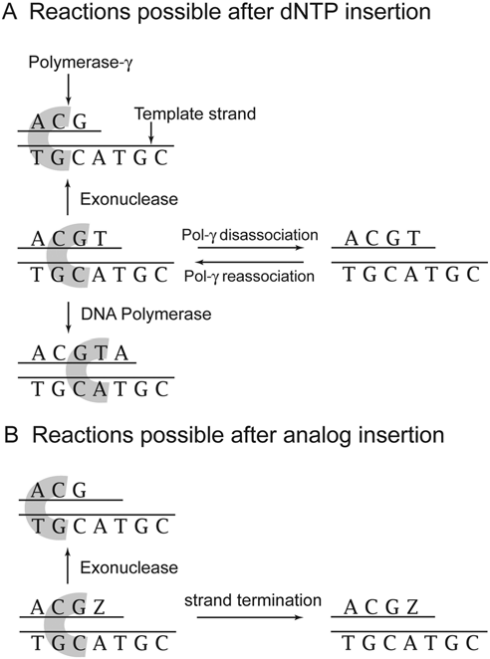
Schematic diagrams of the polymerase-γ reactions in this model. (A) The four reactions possible following a correct incorporation. DNA Polymerase: polymerase-γ adds one nucleotide to the new DNA strand. This nucleotide may be the correct or incorrect (point mutation) base pair for template strand, or a nucleotide analog. Exonuclease: polymerase-γ removes one nucleotide (correct or incorrect match) from the new DNA strand. This is an error correction mechanism. Disassociation: The DNA polymerase can separate from the DNA molecule. Reassociation: The DNA polymerase re-attaches to the DNA molecule after disassociation. (B) The possible reactions after insertion of analog (Z to represent AZT in this example): exonuclease activity or dissociation. Our model does not allow further polymerization or reassociation once an analog is inserted and not removed by an exonuclease reaction.

Our computational model of the mitochondrial DNA replication process is based on the Stochastic Simulation Algorithm [32],[33], a well-known Monte Carlo simulation method for chemical reactions. The model is based on four reactions; DNA polymerase activity, exonuclease activity, disassociation of the polymerase from the DNA, and reassociation of the polymerase with the DNA molecule (Figure 1). In the DNA polymerase reaction pol-γ adds one nucleotide to the new DNA strand. This nucleotide may be the correct or incorrect (point mutation) base indicated by the template strand. In this model this includes the incorporation of nucleoside analog triphosphates. In the exonuclease reaction pol-γ removes one nucleotide from the new DNA strand. This includes the removal of nucleoside analogs from the DNA strand. The exonuclease reaction is an error correction mechanism, as the rate for removal of incorrectly incorporated nucleotides is typically faster than that of correctly incorporated nucleotides. In the disassociation reaction the polymerase separates from the DNA molecule. In the reassociation reaction the polymerase re-attaches to the DNA molecule after disassociation. At each position on the replicating mtDNA strand, pol-γ will randomly undergo one of the first three reactions (polymerase, exonuclease, or dissociation). Which reaction pol-γ undergoes is determined by the probability of each reaction, calculated using the reactions rates and Michaelis-Menten kinetics.

For the two scenarios of a correctly inserted and incorrectly inserted previous nucleotide we have separate sets of kinetic parameters for each of the pol–γ reactions [34]–[36]. These studies have reported an increase in exonuclease and disassociation rates, but a decrease in incorporation rates by pol-γ following an incorrect incorporation. This is included in the simulation model by using two sets of enzyme kinetics parameters, one set for reactions following a correct incorporation and another set for reactions following an incorrect incorporation. Kinetic parameters for the natural nucleotide (dNTP) interaction with pol-γ are available in Text S1. As data regarding the reassociation reaction rate are not available our model assumes that after a disassociation event occurs the reassociation reaction follows, except in the special case discussed immediately below. Since the rate for the reassociation reaction is not available, the time required for that reaction is not calculated in this model. This approximation is not important to our results reported here which focus on strand termination probabilities.

Upon incorporation of an analog into the new DNA strand the next polymerase reaction is blocked. The exonuclease reaction can still occur, removing the analog molecule. However, if a disassociation reaction occurs before the analog can be removed, we assume that reassociation of the DNA polymerase is also blocked and the mtDNA replication event is disrupted resulting in strand termination (Figure 1). There has been some speculation that the drugs, in particular AZT, may be inserted into a replicating mtDNA strand without causing strand termination. In this model we take the conservative assumption that all NRTIs that are inserted in the mtDNA strand and not subsequently removed cause strand termination.

Parameters included in the model for incorporation of each analog by pol- γ were the concentration necessary for binding of 50% of available pol-γ (K_m_), the rate of polymerization (k_pol_),, and the rate of excision (V_exo_) of each analog by pol-γ. The parameters k_pol_ and K_m_ were estimated from the maximum rate of incorporation by pol-γ (k_cat_) and the dissociation constant from pol-γ (K_d_), respectively, obtained under pre-steady state conditions [25]–[27]. A recent publication shows that pyrophosphate release from AZT is uniquely slow during polymerization and that kinetics measured during steady-state conditions give a more accurate k_pol_ estimation [27]. These measurements were carried out on AZT due to the fact that under pre-steady state conditions a decrease in incorporation rate was observed with increased AZT concentration indicating reversible binding. This pattern was not seen with any of the other analogs studied (d4T, 3TC(−), AZT, ddC, ddI, ddA, ABC, TDF, and 3TC(+)) and for this reason reanalysis of the enzyme kinetics for those drugs was not performed in that experiment. Given this continuing evolution in our understanding of the AZT kinetics we carried out two simulations for AZT insertion using the two available published sets of parameters determined under steady-state conditions in the 2007 paper by Hanes and Johnson [27] and pre-steady state conditions published in the 2001 paper by Johnson et al. [25]. We distinguished the results using these two parameter sets as AZT_2001_ and AZT_2007_. These parameter values, as well as those for the other analogs, are given in Table 2.

### Triphosphorylated Mitochondrial Natural Nucleotide (dNTP) Levels

The polymerization reaction rates are functions of the dNTP concentrations. For this calculation we consider three sets of dNTP concentrations, representing high, medium and low concentration conditions. Mitochondrial dNTP levels were estimated following the observations of Rampazzo et al and Ferraro et al [37],[38] (Table 3). The units of picomole of mitochondrial dNTP per mg of mitochondria or picomoles per 10^6^ cells were converted to µM by using an assumed mitochondrial volume of 0.2 femtoliters and density measurements from Pollak and Munn [39]. It should be noted that these density measurements considered mitochondria as discrete entities not taking in to account any change in mitochondrial size due to organelle fission and fusion processes. We use these values only as estimates, in order to define the three categories of dNTP concentrations given below.

**Table 3:** Mitochondrial dNTP concentrations and K_m_ values.

dNTP Level	dATP (µM)	dCTP (µM)	dGTP (µM)	dTTP (µM)
High	22.5	28	19.5	26
Medium	1.675	1.644	0.47	0.76
Low	0.1675	0.1644	0.047	0.076
K_m_ with polymerase-γ^a^	0.8	0.9	0.8	0.6

a From reference [36].

#### High dNTP levels

As an estimate for the natural nucleotide concentrations within mitochondria of actively dividing cells, concentrations of natural nucleotides in mitochondrial pools from a cycling cell culture of 3T3-L1TK1+ (a mouse fibroblast line) were used. [38]. The units of dNTP measurement were converted to µM units by estimating the volume of a 3T3-L1 cell from images in Friis et al. [40].

#### Medium dNTP levels

As an estimate of the natural nucleotide concentrations within mitochondria of resting or slowly dividing cells values for rat liver cells were used [41].

#### Low dNTP levels

A third set of simulations were carried out using natural nucleotide levels at 1/10th those estimated for the liver cells. This is meant to represent the low dNTP concentrations in postmitotic cells.

### Simulation Sets

Vertebrate mitochondrial DNA has a highly asymmetric G content. The low-G strand is labeled the light strand, with the complement strand called the heavy strand. Sets of simulations were carried out separately for the light strand sequence (NCBI, gi 17981852) and the heavy strand sequence. On each template three separate simulation sets were carried out using the high, medium, or low natural nucleotide concentrations described above and varying the concentration of activated triphosphorylated analog. Each simulation was repeated 1000 times. The number of simulated mtDNA strand replications ending in a strand termination event (caused by a nucleoside analog incorporation) was recorded. The concentrations of the four dNTP pools and the activated analog were held constant throughout each simulated replication.

## Results

The purpose of this study was to explore the plausibility of the pol-γ hypothesis by calculating the probability of insertion of the nucleotide analog into the replicating mtDNA strand, leading to strand termination. We constructed a model based on the mitochondrial genome length and sequence, mitochondrial dNTP concentrations, and the measured enzyme kinetics of pol-γ. With this model we simulated mitochondrial DNA replication in the presence of different analogs determining the dose response curves and the IC_50_ values of DNA termination for each drug. The simulation results were compared to reports of mitochondrial toxicity, and specifically to reports of mtDNA depletion.

### Dose Response Curves and IC_50_ Values

**Figure 2:**
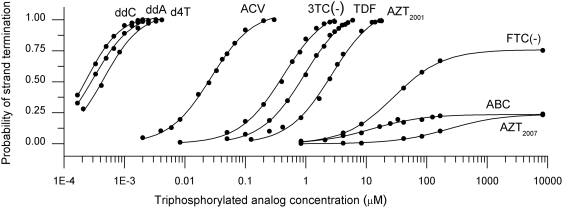
The dose-response curves for incorporation probability of analogs approved for treatment. Circles are the probability of strand termination calculated from a set of 1000 simulations for each point and the curves are dose-response curves fit to the data points. There is a large difference of incorporation probability between the di-deoxy drugs (ddC, ddI, and d4T) and other approved anti-retrovirals (3TC, TDF, AZT, ABC, and FTC). The anti-herpes drug, acyclovir, falls in the middle of these two extremes, but shows little mitochondrial toxicity in clinical use possibly due to the fact it is dependent upon viral proteins for activation. AZT_2001_ probabilities were determined using kinetic parameters from reference [25] and AZT_2007_ probabilities were determined using newly reported kinetic parameters from reference [27].

By measuring the probability of strand termination in the simulation as a function of the activated drug concentration, dose response curves for each drug were calculated. Figure 2 shows the dose response curves obtained for the strand termination probability of each clinically approved analog as a function of the analog mitochondrial concentrations. The concentration at which these dose response curves passed 50% defined the IC_50_ values for each activated drug (Table 4). In our model, replication was terminated once an analog was inserted and failed to be removed by exonuclease activity. Based on these simulated IC_50_ values the list of analogs in the order of decreasing probability of mtDNA strand termination on the light strand was: ddC = ddA = d4T>FTC(+)>3TC(+)>ACV>ddI>3TC(−) = TDF> = AZT_2001_>>FTC(−)>ABC = AZT_2007_ in which “>>” indicates a 10 fold difference or more, “>” indicates a 2 to 10 fold difference and “ = ” indicates a less than 2 fold difference. Note that ddA is the activated form of ddI. Of this list only d4T, ddI, ddC, 3TC(−), TDF, AZT, ABC, ACV and FTC(−) are approved for therapeutic use. The IC_50_ list showed that the “di-deoxy drugs”, meaning ddC, d4T, and ddA, had the highest probability of causing mtDNA strand termination during replication while FTC(−), ABC and AZT_2007_ showed the least. Of those drugs approved for HIV treatment there was an observed difference of more than 800 fold between the di-deoxy drugs (ddC, ddA, and d4T) and other approved drugs (3TC, TDF, AZT, ABC, and FTC(−)) in the activated drug concentration necessary for 50% probability of mtDNA strand termination.

**Table 4:** IC_50_ values for light strand termination calculated from the simulation.

Analog	IC_50_Values (µM) with High dNTP	IC_50_ Values (µM) with Medium dNTP	IC_50_ Values (µM) with Low dNTP	Reported mtDNA Depletion	Citations for mtDNA Depletion
ddC-TP	3.42×10^−4^	2.38×10^−4^	5.80×10^−5^	Yes	[8],[11]
ddA-TP	4.42×10^−4^	2.89×10^−4^	7.47×10^−5^	Yes	[8],[11]
d4T-TP	7.92×10^−4^	4.65×10^−4^	9.76×10^−5^	Yes	[8],[11]
FTC(+)-TP	5.90×10^−3^	4.20×10^−3^	9.80×10^−4^	No data	NA
3TC(+)-TP	3.54×10^−2^	2.26×10^−2^	6.30×10^−3^	No data	NA
Acyclovir-TP	6.78×10^−2^	2.58×10^−2^	3.81×10^−3^	No data	NA
ddI-TP	0.25	0.17	4.60×10^−2^	No data	NA
3TC(−)-TP	0.56	0.40	8.40×10^−2^	No	[11],^55^
TDF-TP	1.18	0.87	0.22	No	[68],[69]
AZT_2001_-TP	3.95	2.38	0.50	No	[9], [11], [18], [19], [56]–[62]
FTC(−)-TP	778.9	58.59	7.57	No	
ABC-TP	NA	NA	8.10	No	[15]
AZT_2007_-TP	NA	NA	NA	No	[9], [11], [18], [19], [56]–[62]

Analogs are listed in order of increasing IC_50_. IC_50_ values are calculated for three different sets of mitochondrial dNTP levels; high, medium and low, as defined in Table 3. NA, not applicable.

The only difference seen in the simulation of heavy strand replication (Text S1) was that acyclovir had a slightly higher probability of termination than 3TC(+) and ABC had approximately equal probability of termination as FTC(−). Since there was little difference in the results for the two strands of the mtDNA molecule, we concentrated on results from the light strand. For the readers' convenience in interpreting these IC_50_ values, reported ranges of intracellular concentrations [42]–[48] for activated nucleoside analog drugs measured in peripheral blood mononuclear cells in patients are given in Table 5. Where necessary, values were converted to units of µM using the conversion of Kewn et al [45]. However it should be kept in mind that these concentrations are intracellular values, not the concentration values in the mitochondria which may be different.

**Table 5:** Reported intracellular concentrations of the activated (tri-phosphate) form of the nucleoside and nucleotide analogs, measured in peripheral blood mononuclear cells in patients.

Activated Analog	Intracellular Concentration Range (mM)
3TC-TP	35.4–51.2
FTC(−)-TP	0.40–4.50
AZT-TP	0.84–1.2
ABC-TP	0.04–0.75
TDF-TP	0.27–0.39
ddA-TP	0.013–0.078
d4T-TP	0.016–0.082
Acyclovir-TP	Not available
ddC-TP	Not available

### Abacavir, Emtricitabine, and Zidovudine_2007_

For most analogs, the simulated dose response curve increases to 100% probability of strand termination if the analog concentration is raised high enough. AZT_2007_, ABC and FTC(−) behaved differently from the other analogs in that they reached the point of saturation below 100% probability of strand termination (Figure 2), and in some cases the strand termination probability saturated below 50%, meaning that no IC_50_ values could be defined in those cases (the blank entries in Table 4). These three analogs interact so poorly with pol-γ that over the finite length of the mtDNA sequence (approximately 16,600 base pairs) these analogs have too few chances to incorporate into the growing mtDNA strand for the probability of strand termination to approach 100%, even with very large concentrations of the activated drug in the mitochondrion.

When the recently revised steady-state derived parameters for k_cat_ and K_m_ for AZT [27] were used in the simulation, AZT_2007_ did not reach a 50% probability of strand termination in the presence of normal to high dTTP levels, instead saturating at a 23% probability. This grouped AZT_2007_ with ABC as having the least probability of causing termination of the replicating mtDNA.

### Specificity Constant

**Figure 3:**
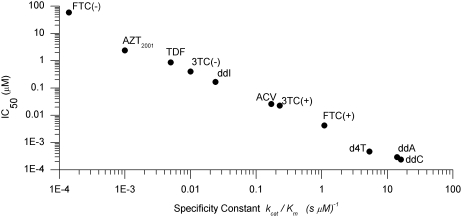
The relationship between the IC_50_ values and the specificity constant, k_cat_/K_m_. The specificity constant is a measurement of direct interaction with polymerase-γ often used as prediction for analog incorporation. The IC_50_ values are a more direct measure of incorporation probability taking exonuclease activity and other features into account. This relationship shows that the specificity constant is a useful proxy for incorporation probability.

A common measurement for the relative likelihood of strand termination by each analog is the specificity constant [25] determined by the ratio k_cat_/K_m_ for the incorporation of an analog by pol-γ. This is a common measurement used for predicting the discrimination of analogs by pol-γ over the natural nucleotide substrate [25]–[28]. The drawback in taking this measurement of direct interaction with pol-γ as a predictor for the actual incorporation into the replicating mtDNA strand is that the specificity constant does not consider exonuclease activity, mitochondrial dNTP levels, nor strand length, all of which can affect the probability that an analog will be incorporated. All of these factors of the system are integrated into our computational model and the resulting IC_50_ values. Previous studies [25]–[28] provide a list of increasing specificity constants of: ddC>ddA>d4T>>ACV>3TC(−)>TDF>AZT>>ABC = FTC(−). The order of this list agreed quite well with our list given above based on simulated mtDNA strand termination. This agreement validates the use of k_cat_/K_m_ values as an appropriate proxy for the relative probability of incorporation of these NRTIs by pol-γ. A quantitative comparison between the specificity constant and our calculated IC_50_ for strand termination is given in Figure 3.

### Removal of Nucleoside Analogs from the mtDNA

The very low exonuclease reaction rate for each analog is the primary reason why the specificity constant serves as a reasonable prediction of mtDNA strand termination. The exonuclease reaction rates used in this study were taken from Johnson et al. [25]. Low excision rates for NRTIs have also been documented in the case of ddC [49] and using yeast mtDNA polymerase with ddC and AZT [50]. However, 3TC has a non-negligible measured exonuclease rates (Table 2) [25]. Whenever an analog is inserted into the DNA strand, our pol-γ model assumed that only the exonuclease and pol-γ dissociation reactions can occur. Based on this model, in Table 6 we give the predicted probability P_exo_ of the analog removal
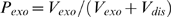
where V_exo_ is the rate of exonuclease reaction for the analog and V_dis_ is the rate of disassociation of the polymerase. To test these predictions, we carried out a set of simulations with the analog exonuclease reactions removed. The ratio of the IC_50_ value in the full model to the IC_50_ value in the exonuclease deficient model was in very good agreement with the 1-P_exo_ values (Table 6). As predicted, only the two 3TC forms showed significant effects from the removal of the analog exonuclease reaction. Even in these cases the effect of the exonuclease reaction only shifted the IC_50_ value by a factor of 2 or less.

**Table 6:** The effect of the exonuclease reaction for each nucleoside or nucleotide analog, in order of increasing V_exo_.

Analog	V_exo_ (1/s)	P_exo_	1-P_exo_	IC_50_/IC_50_(exo-)
ddC	0.00002	0.00040	0.9996	1
d4T	0.0004	0.0079	0.9921	0.98
AZT_2001_	0.0004	0.0079	0.9921	1.038
AZT_2007_	Not reported	-	-	-
ddA	0.0005	0.01	0.99	1.086
ddI	0.0007	0.014	0.986	1.059
TDF	0.0007	0.014	0.986	0.942
ABC	0.0016	0.031	0.969	-
Acyclovir	0.0021	0.040	0.96	0.919
FTC(−)	0.0048	0.088	0.912	0.964
FTC(+)	0.0048	0.088	0.912	0.867
3TC(−)	0.015	0.23	0.77	0.725
3TC(+)	0.02	0.29	0.71	0.69

### Effects of Multiple Nucleoside Analogs

**Figure 4:**
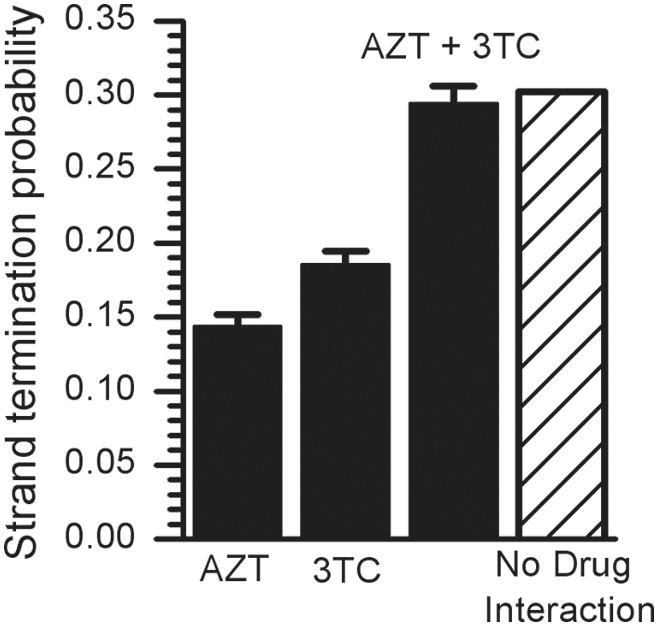
The computed probability of strand termination for an AZT and 3TC combination. Computed probabilities are shown for AZT-TP alone (at 0.5 µM), 3TC-TP alone (at 0.1 µM) and the combination of both drugs at those concentrations (solid bars). The error bars represent standard deviations from 10 repeated sets of 1000 simulations. Also shown is the predicted probability of strand termination for the combined drugs (hatched bar) with the assumption of no interaction between the two drugs, calculated from the equation given in the text.

The current therapy for HIV infections involves a combination of nucleoside analog drugs, along with another class of drug such as a protease inhibitor. It has been reported that combining nucleoside analogs increases toxicity [51],[52]. The pol-γ model is a series of reactions occurring as the DNA polymerase moves along the template strand. At each position on the DNA strand different nucleoside analogs would be able to be incorporated into the DNA strand. For example, AZT triphosphate molecules would only have a reasonable rate of incorporation opposite an A on the template strand, while 3TC triphosphate molecules would only have a reasonable rate of incorporation opposite a G on the template. Considering this, it is unlikely that there could be a combined effect of two analogs of different nucleosides on strand termination through the pol-γ interaction alone. To test this, we modeled the effects of two analogs, AZT and 3TC, separately and in combination (Figure 4). The combination of AZT and 3TC has been shown to have enhanced toxicity [51],[52], though neither of these two studies found any significant mtDNA depletion associated with this toxicity. If we define P_AZT_ as the probability of strand termination from a given concentration of AZT triphosphate and P_3TC_ as the probability from a given 3TC triphosphate concentration, then the combination of the two drugs should result in a strand termination probability of
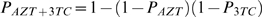



This equation assumes there is no interaction between the two nucleoside analog drugs (this is known as the Webb fractional effect [53]). Note that P_AZT+3TC_ is here defined as one minus the probability that neither AZT nor 3TC independently cause strand termination. A set of 1000 simulations was repeated 10 times, using the medium dNTP concentrations defined in Table 3, and mean and standard deviations for the probabilities P_AZT_, P_3TC_ and P_AZT+3TC_ were measured. The results for P_AZT+3TC_ were consistent with the probability expected assuming no interaction between the two drugs (Figure 4). This indicates that any synergistic effects of multiple NRTIs on mitochondrial toxicity are not consequences of direct strand termination. Alternative explanations for synergistic effects may include competitive inhibition of deoxynucleotide phosphorylation, which is outside the limits of this computational model.

## Discussion

Our simulated IC_50_ values of mtDNA strand termination for AZT-TP, CBV-TP (ABC), FTC(−)-TP and TDF-DP were approximately 1,000-fold or more higher than those of the di-deoxy drugs. This agrees with the lack of observed mtDNA depletion in liver, fat, and PBMC samples from patients on regimens comprised of NRTIs that are not di-deoxy analogs [8],[9],[11]. The herpes drug Acyclovir (ACV) fell between the di-deoxy drugs and the others in terms of probability of causing mtDNA strand termination. ACV is not associated with mitochondrial toxicity clinically. This is believed to be due to the fact that the drug must be activated by a viral-encoded kinase [54]. As herpes replication tends to occur in waves with long latent periods, this may not lead to the long term effects on mitochondria seen with NRTI use for HIV treatment.

### The Di-deoxy Drugs

The di-deoxy NRTIs (ddC, ddA, and d4T) showed the greatest risk of strand termination in our simulations, indicated by their predicted low IC_50_ values. This agrees with previous studies showing they are the NRTIs most associated with mtDNA depletion *in vitro* [14]–[16],[18],[19],[55] and mitochondria-related toxicities clinically placing them as alternative drugs in the federal guidelines [1]. In both muscle and subcutaneous fat biopsies of HIV+ patients, mtDNA levels were significantly lower in those on di-deoxy drug regimens as opposed to those on non-di-deoxy NRTI regimens [8],[11]. Even though the toxic side effects of di-deoxy drugs are well known and the *in vitro* effects on tissue mtDNA levels of these drugs are in agreement with our simulation results, the very low IC_50_ values for these drugs of approximately 3×10^−4^ µM warrant discussion. The low IC_50_ value for ddC is in agreement with findings that this drug is not readily metabolized in the cell to its active form [16],[47], implying that the concentrations of the activated drug in the cell may be quite small. There is evidence, however, that d4T and ddI are activated to a significant degree as the concentration of their triphosphorylated forms in patient peripheral blood mononuclear cells are above the predicted IC_50_ values by approximately 100-fold [43],[44] (Table 5, note that the experimentally measured value is the activated drug concentration in the cytoplasm, not in the mitochondria). Given that d4T and ddI are still recommended drugs for HAART [1] they are obviously tolerable to a large number of patients who do not experience the serious side-effects of lactic acidosis and neuropathy. One plausible explanation for this tolerance in the face of the striking affinity of these drugs for pol-γ is that there exists a significant barrier to di-deoxy drug entry into the mitochondrion or drug activation within the mitochondrion allowing activated di-deoxy drug mitochondrial concentrations to remain low in the majority of patients treated with these drugs. We know of no reports of measured levels of the triphosphate form of these di-deoxy drugs within mitochondria.

### AZT

The experimental data indicates that AZT interacts poorly with pol-γ as shown by the high K_m_ and low k_cat_ values (Table 2) for this drug. An explanation for the slow rate of incorporation of AZT was recently published [27]. AZT demonstrates unusually slow pyrophosphate release upon incorporation by pol-γ rendering polymerization readily reversible even upon binding to the template∶primer molecule. In the cases of natural nucleotides this subreaction is fast enough to be considered negligible indicating that pre-steady state k_pol_ values are a good approximation for k_cat_. Yet, in the case of AZT, this slow pyrophosphate release rate has a significant effect on k_cat_ so that a measurement of k_pol_ during steady-state conditions is more appropriate for estimating k_cat_. The k_pol_ and K_d_ determined in the recent Hanes and Johnson study [27] with steady-state conditions, that theoretically take the slow pyrophosphate release rate in to account, indicate a k_cat_ 100-fold lower than that determined from the k_pol_ calculated under pre-steady state conditions [25]. We carried out the simulation for both sets of parameters separately and in both cases AZT shows a poor probability of mtDNA strand termination (Figure 2). The IC_50_ values generated by this study show that AZT should not be toxic through mtDNA strand termination as it has a higher IC_50_ value than 3TC(−) and TDF, neither of which are associated with mitochondrial toxicity.

The low probability of strand termination by AZT is supported by the fact that although AZT has consistently been associated with positive markers for mitochondrial toxicity, substantial evidence exists that the extent of AZT-induced mitochondrial toxicity is disproportional to the amount of mtDNA depletion it causes. The analogs ddC, d4T and ddI (activated to ddA) cause significantly more mtDNA depletion and decreased protein subunit expression of various electron transfer chain proteins with essential subunits encoded in the mtDNA, as would be expected from their increased interaction with pol-γ compared to other analogs (Figure 2). Yet AZT still manages to demonstrate a cytotoxicity that is equal to or greater than ddA, ddC, and d4T at comparable concentrations in various studies. In human liver and cardiac muscle cells incubation with AZT lead to cytotoxicity and increased lactate levels with no sign of mtDNA depletion [18],[56],[57]. Similar results are seen in blood cells and adipose cells [19],[58],[59]. Szabados et al. [60] showed significant toxic effects on cardiac muscle cells including increased ROS, abnormal mitochondrial structure, and decreased ATP/ADP ratio after two weeks of exposure of cells in medium with no effects on mtDNA levels. In fact, AZT is actually associated with slight increase in mtDNA levels in cell culture [61],[62], PBMCs [9], and liver tissue samples [11]. Our model, however, does not address subreactions that influence pol-γ binding of the analog, meaning our results cannot disprove the possibility that AZT toxicity is due to deactivating pol-γ either through irreversible binding or induction of a conformational change in the enzyme. However, the high K_d_ determined by both Hanes and Johnson and Johnson et al. [25],[27], along with the cited studies showing toxicity independent of mtDNA depletion, make this an improbable mode of toxicity. It is our conclusion that based on the measured kinetic coefficients of AZT with pol-γ that AZT toxicity is not dependent upon mtDNA strand termination. Indeed, various pol-γ independent hypotheses have been proposed for AZT mitochondrial toxicity. These include inhibition of the enzymes of the mitochondrial salvage pathway causing nucleotide pool imbalances [63], binding to ADP-ATP translocator [64], and direct inhibition of components of the electron transport chain [65].

### TDF

Tenofovir is associated with renal dysfunction without significant mtDNA depletion [66],[67]. In a retrospective study of HIV positive patients taking TDF and those not taking TDF, no significant differences in mtDNA levels of kidney biopsies were observed [68]. Similarly, in human renal proximal tubule cells [69], TDF was not associated with cytotoxicity, mtDNA depletion, or COII mRNA depletion. In our simulations mitochondrial TDF triphosphate IC_50_ values were in the range 0.2 to 1.2 µM, depending on the natural dNTP levels. Since these concentrations are not unusually high, our conclusion is that Tenofovir might be able to cause some moderate mtDNA depletion, depending on how well the activated drug is concentrated within the mitochondrion.

### Conclusions

A number of hypothesis with supporting evidence have been proposed for NRTI toxicity experienced during HAART. Possible pol-γ mediated pathways include the direct inhibition of pol-γ by NRTI-triphosphate without incorporation of the analog; chain termination by incorporation of NRTI triphosphate into mtDNA; and incorporation without chain termination of the analog-triphosphate allowing it to remain as a point mutation in mtDNA. Our model only addresses the case of chain termination. There is not enough data on the subreactions that comprise analog binding to pol-γ for this model to explore the possibility that some analogs cause toxicity through inhibition of the pol-γ enzyme directly, either by irreversible binding or induction of conformational change, as opposed to strand termination.

The specificity constant, k_cat_/K_m_, [25] is commonly used as an approximate indicator of mitochondrial toxicity through strand termination of mtDNA. Before this model, this has been a bit of a leap as the specificity constant does not take genome length, exonuclease activity, nor dNTP concentration into account, and no direct predictions or measurements of strand termination probabilities have previously been given. We fill this gap in our understanding by providing a model that includes all of these factors and that predicts strand termination probabilities. The consistence between our simulation model results and the qualitative ordered list of NRTI drug toxicity based on the specificity constant is a validation of the model results. However, the simulation model goes far beyond the specificity constant by predicting IC50 values and quantitative dose-response curves (Figure 2) for these drugs. Furthermore, the specific definition of strand termination used in this model raises the hypothesis that dissociation of polymerase-γ after an NRTI is incorporated into the mtDNA strand is a critical step in strand termination. In this particular model we chose to define strand termination as the dissociation of polymerase-γ after the incorporation of an NRTI, under the assumption that re-association of the polymerase after the NRTI could not occur. Of course, it is possible that in-vivo there may be other currently unknown factors which may alter the polymerase-γ dissociation kinetics (or any other kinetics for that matter) from the measured values. If our assumption that pol-γ re-association is blocked after NRTI incorporation was changed, and re-association of the polymerase was to be allowed, then more exonuclease events of the NRTIs would occur. However, it is not clear to us then what the definition of “strand termination” would be since the exonuclease activity would eventually remove all incorporated NRTIs given enough time. Johnson et al [25] used that assumption, where all NRTIs incorporated into the mtDNA were eventually removed by exonuclease activity, to define a toxicity index based on a calculation of the amount of additional time required for these NRTI exonuclease events. Based on this definition of a toxicity index, Johnson et al [25] also defined an ordered list of NRTI drug toxicity which was similar to our list and similar to the lists based on the specificity constants. An important use of any computational model is to raise questions for further experimental study. This simulation raises the following questions. Is strand termination defined by the dissociation of the polymerase after insertion of an activated NRTI? If not, what is the proper definition of strand termination?

NRTI toxicity appears perplexingly specific to cell type [1] and the mechanism for this tissue specificity is currently unclear. As natural nucleotide concentrations within the mitochondrion can differ greatly across cell types, we sought to observe how incorporation of NRTIs may differ in the presence of varying mitochondrial dNTP levels, which we broke down into three sample categories; high dNTP levels, medium dNTP levels and low dNTP levels (Table 3). Although the IC_50_ values for strand termination measured in this simulation did depend on the concentrations of the natural nucleotide triphosphates, the relative ordering of the nucleoside analogs by IC_50_ was the same for all three dNTP conditions (Table 4). The simulations made with low dNTP concentrations, representing post-mitotic tissues, did have lower IC_50_ values, consistent with a greater sensitivity of these tissues to damage by nucleoside analog drugs. However, these results would not explain why some tissues are susceptible to toxicity from a particular analog. We have limited this simulation model to the activity of the mitochondrial DNA polymerase acting on the tri-phosphate form of the four natural deoxyribonucleosides and the tri-phosphate form of the drugs. Since the tissue dependence of the toxicity of the drugs was not reproduced in this model, this implies that the source of this tissue dependence lies outside the bounds of this particular model. This includes the possible interference of the various phosphate states of the drugs with the metabolism within the mitochondria that produces the natural deoxyribonucleoside tri-phosphates, potentially altering the relative levels of the four natural dNTPs.

Although mitochondrial toxicity from NRTIs is common, the more severe forms of this toxicity are certainly not universal. Current research is revealing that the gene for polymerase-γ is the site of a large number of mutations and polymorphisms that alter its enzyme kinetics and function [70]–[73]. The natural variability in this crucial gene may be an important source of the individual variation in the susceptibility of patients to this toxicity, and perhaps to the phenotypic variation which occurs. Although the interaction of nucleoside analogs with polymerase-γ has been recognized for almost 15 years now [6], we still know surprisingly little about the levels of activated drugs within mitochondria [63] or about the transport mechanism by which these drugs enter the mitochondrion [74],[75].

## Supporting Information

Text S1 Supplemental tables of model parameter values and additional results
(0.03 MB PDF)
