# Supplementary material for: An Analysis of Enzyme Kinetics Data for Mitochondrial DNA Strand Termination by Nucleoside Reverse Transcription Inhibitors
Source: PLoS Comput Biol. 2009 Jan 9;5(1):e1000261. doi: 10.1371/journal.pcbi.1000261 (PMC2603287; doi:10.1371/journal.pcbi.1000261)
Supplement: Text S1 — Supplemental tables of model parameter values and additional results (0.03 MB PDF) [file pcbi.1000261.s001.pdf]

An Analysis of Enzyme Kinetics Data for Mitochondrial DNA Strand Termination by Nucleoside Reverse Transcriptase Inhibitors

Katherine V. Wendelsdorf[1], Zhuo Song[1], Yang Cao[2] and David C. Samuels[1,3*]

1 Virginia Bioinformatics Institute, Virginia Polytechnic Institute and State University, Virginia, USA, **2** Department of Computer Science, Virginia Polytechnic Institute and State University, Virginia, USA, **3** The Center for Human Genetics Research, Department of Molecular Physiology and Biophysics, Vanderbilt University Medical Center, Nashville, Tennessee, USA

Supplemental Information: Model parameter values.

TABLE S1**.** Kinetic parameters k_{cat} (1/s) for base pairings when previously inserted nucleotide is correct (a Watson-Crick pair).

**Base pairings**	**T**	**G**	**C**	**A**
**T**	25	0.08	0.1	0.0036
**G**	0.012	37	0.003	0.1
**C**	0.16	0.066	43	0.042
**A**	0.013	1.16	0.038	45

Values were taken from Lee and Johnson (45) in which the previous base pair is A:T

TABLE S2**.** Kinetic parameters K_m (μM) for base pairings when previously inserted nucleotide is correct.

**Base pairings**	**T**	**G**	**C**	**A**
**T**	0.6	800	540	25
**G**	180	0.8	140	160
**C**	200	150	0.9	250
**A**	57	70	360	0.8

Values were taken from Lee and Johnson (45) in which the previous base pair is A:T

[*] David Samuels, Virginia Bioinformatics Institute, Bioinformatics I, Washington Street Blacksburg, VA 24061-0477 Tel: (540) 231-8999, Fax: (540) 231-2606, email: david.samuels@chgr.mc.vanderbilt.edu

TABLE S3. Estimated k_{cat} (1/s) kinetic parameters for base pairings when previously inserted nucleotide is a point mutation (a non-Watson-Crick pair).

**Base pairings**	**T**	**G**	**C**	**A**
**A**	0.52	0.00052	0.00052	0.00052
**C**	0.00052	0.52	0.00052	0.00052
**G**	0.00052	0.00052	0.52	0.00052
**T**	0.00052	0.00052	0.00052	0.52

The k_{cat} values for insertion of C onto G measured by Johnson and Johnson (40-41) where the previous base pairing is a non-Watson-Crick base pairing of T:T as opposed to A:T. This value was assumed an approximation of those for other Watson-Crick pairings based on similarities of k_{cat} values in Table A1 for Watson-Crick base pairings (A:T, C:G). Values for non-Watson-Crick base pairing were made based on the observation from TABLE S1 that k_{cat} values are approximately 1000 times less for non-Watson-Crick base pairing than for Watson-Crick base pairing.

TABLE S4. Estimated K_m (μM) kinetic parameters for base pairings when previously inserted nucleotide is a point mutation.

**Base pairings**	**T**	**G**	**C**	**A**
**A**	404	40400	40400	40400
**C**	40400	404	40400	40400
**G**	40400	40400	404	40400
**T**	40400	40400	40400	404

The K_m values for insertion of C onto G measured by Johnson and Johnson (40-41) where the previous base pairing is a non-Watson-Crick base pairing of T:T as opposed to A:T. This value was assumed an approximation of those for other Watson-Crick pairings based on similarities of K_m values in Table S2 for Watson-Crick base pairings (A:T, C:G). Values for non-Watson-Crick base pairing were made based on the observation from TABLE S2 that K_m values are approximately 100 times greater for non-Watson-Crick base pairing than for Watson-Crick pairing.

TABLE S5. Exonuclease and Disassociation by polymerase-gamma reaction rates when previous base pair is a Watson-Crick pairing vs. non Watson-Crick.

**Previous base pairs**	**Exonuclease rate (1/s)**	**Disassociation rate (1/s)**
**Watson-Crick pair**	0.05	0.02
**Non-Watson-Crick pair**	0.4	0.2

Supplemental Information: Other results

TABLE S6. IC_{50} values (μM) for the strand termination on the mtDNA heavy strand. NA – not applicable, meaning that the probability of strand termination never exceeded 50%.

	**IC_{50} values (μM)**		
**Analog**	**High dNTP levels**	**Medium dNTP levels**	**Low dNTP levels**
**d4T-TP**	$6.75x10^{-4}$	$3.60x10^{-4}$	$7.50x10^{-5}$
**ddA-TP**	$5.20x10^{-4}$	$4.00x10^{-4}$	$9.80x10^{-5}$
**ddC-TP**	$8.90x10^{-4}$	$5.80x10^{-4}$	$1.30x10^{-4}$
**FIAU-TP**	$1.44x10^{-3}$	$8.30x10^{-4}$	$1.60x10^{-4}$
**FTC(+)-TP**	$1.40x10^{-2}$	$1.00x10^{-2}$	$2.20x10^{-3}$
**acyclovir-TP**	$2.85x10^{-2}$	$1.14x10^{-2}$	$1.79x10^{-3}$
**3TC(+)-TP**	$8.8x10^{-2}$	$5.60x10^{-2}$	$1.36x10^{-2}$
**ddI-TP**	0.33	0.23	$5.75x10^{-2}$
**3TC(-)-TP**	1.50	1.00	0.19
**TDF-TP**	1.45	1.06	0.26
**AZT_{2001}-TP**	3.00	1.94	0.37
**AZT_{2007}-TP**	NA	NA	143
**CBV-TP**	NA	NA	2.22
**FTC(-)-TP**	NA	NA	30.9
